# Ethylhexadecyldimethylammonium bromide, a quaternary ammonium compound, controls inflammatory response through NRF2 pathway in a human immortalized keratinocyte cell line

**DOI:** 10.3389/ftox.2023.1132020

**Published:** 2023-04-06

**Authors:** Lise Aubry, Romain Vallion, Sara Salman, Marie-Hélène Damiens, Pierre-Jacques Ferret, Saadia Kerdine-Römer

**Affiliations:** ^1^ Université Paris-Saclay, Inserm, Inflammation microbiome immunosurveillance, Orsay, France; ^2^ Safety Assessment Department, Pierre Fabre Dermo Cosmétique, Toulouse, France

**Keywords:** quaternary ammonium, irritation, sensitization, Nrf2, skin inflammation, ethylhexadecyldimethylammonium bromide, keratinocyte, NF-kB

## Abstract

Many everyday products contain quaternary ammonium compounds (QAC) and some of them are known to be skin irritants such as benzalkonium chloride. Others, such as didecyldimethylammonium chloride, have been shown to cause allergic contact dermatitis. Ethylhexadecyldimethylammonium bromide (EHD) is a QAC for which sensitization potential is not clearly known. Therefore, we have studied its mechanism in human keratinocytes (KC), the main cells of the epidermis. We used the well-described human KC cell line KERTr exposed to EHD, cinnamaldehyde (CinA), a well-known skin sensitizer, and a mixture of both. Since chemical sensitizers are known to activate the transcription factor nuclear factor (erythroid-derived 2)-like 2 (NRF2), leading to cellular detoxification and suppressed proinflammatory cytokines, protein or mRNA expression of NRF2 pathway-related enzymes and pro-inflammatory cytokines were investigated by Western blot and RT-qPCR. The activity of the NRF2 pathway on inflammation was studied by RT-qPCR in *NRF2*-invalidated KERTr cells. We showed that EHD cannot induce the NRF2 pathway, unlike contact sensitizers like CinA. EHD triggers an inflammatory response by inducing the mRNA expression of pro-inflammatory cytokines such as IL-1β or IL-6. Moreover, mixing EHD and CinA inhibits the effect of CinA on NRF2 expression and mitigates the inflammatory response induced by EHD alone. EHD treatment of KERTr cells in which *NRF2* has been invalidated showed an exacerbation of the inflammatory response at the transcriptional level. Hence, EHD may elicit an inflammatory response in KC *via* the NF-κB pathway, which could lead to irritation when applied to the skin. This inflammation is negatively controlled by the basal activity of the NRF2 pathway.

## Introduction

Quaternary ammonium compounds (QAC) are synthetic chemicals commonly found in many products such as cosmetics (*e.g.,* facial cleansers, Sun protection creams or lotions, baby lotions, moisturizers, hair conditioners, hair color, make-up) and biocides due to their antimicrobial, surfactant, and preservative properties. They contain at least one NH_4_^+^ group linked to a hydrocarbon chain and three other radicals, *e.g.*, methyl or benzyl groups (Buffet-Bataillon et al., 2012). Benzalkonium chloride^1^ is the most investigated QAC and is recognized as a strong skin irritant. It may also act as an allergen since it has been implied in some hypersensitivity reactions, including occupational asthma and skin-related symptoms (Bernstein et al., 1994). Whereas sensitization to benzalkonium chloride can occur, it remains quite rare (Dao et al., 2012). Didecyldimethylammonium chloride^2^ (DDAC) is a more recent QAC that has been identified as a triggering event of skin inflammation such as allergic contact dermatitis (ACD) (Dejobert et al., 1997; Mowitz and Pontén, 2015).

ACD is a common disease mediated by T-cell. The sensitization phase is induced by contact sensitizers, low molecular weight compounds called haptens. They penetrate the epidermis and form an immunogenic complex with a skin protein (Karlberg et al., 2008). This complex induces an innate immune response in the skin by activating dendritic cells (DC), which have an essential role in initiating the antigen-specific primary immune response (Martin, 2012). They migrate from the skin to the draining lymph nodes, where they present the haptenized protein to T-cell leading to an adaptive response (Karlberg et al., 2008).

Keratinocytes (KC) constitute the major cellular population in the epidermis (Kanitakis, 2001). They respond to inflammatory stimuli through a wide variety of cytokines and pathogen-associated molecular pattern (PAMP) receptors. These cells are also a source of many inflammatory mediators which could initiate a skin inflammatory response and promote the recruitment of inflammatory cells such as neutrophils in the skin (Jiang et al., 2020). After exposure to contact sensitizer, KC release danger signals such as pro-inflammatory cytokines. These signals can be recognized by receptors on the surface of innate immune cells, such as Toll-like receptors (TLR) (Juráňová et al., 2017). Then, some cytokines such as interleukine-1β (IL-1β) and tumor necrosis factor-α (TNF-α) produced by KC have a role in the maturation and migration of DC during the sensitization phase (Cumberbatch et al., 1997; 2001). Haptens have been shown to induce the NF-κB (Nuclear factor kappa-light-chain-enhancer of activated B-cell) pathway involved in inflammation (Tak and Firestein, 2001; Christensen and Haase, 2012). Under physiologic conditions, NF-κB is a complex composed of the proteins p65 and p50. It is sequestered in the cytoplasm by its inhibitor, IκB. After stimulation of TLR by, *e.g*., pathogens, IκB is phosphorylated, ubiquitinated and degraded by the proteasome (Juráňová et al., 2017). Then, p65 is phosphorylated (Zhong et al., 1997). These events lead to the translocation of the complex into the nucleus, where it notably induces the transcription of chemokines, cytokines such as IL-6, IL-8, TNF-α and proteinases (Juráňová et al., 2017).

Moreover, haptens are electrophilic compounds and generate oxidative stress in the epidermis, activating the NRF2 (Nuclear factor E2-related factor 2) pathway (Christensen and Haase, 2012; Helou et al., 2019). NRF2 is a transcription factor of its target genes like *HMOX1* encoding HO-1 (Heme oxygenase-1) and *NQO1* (NAD(P)H Quinone Dehydrogenase 1). Briefly, under basal conditions, two proteins of KEAP1 (Kelch-like ECH-associated protein 1) hold the protein NRF2 in the cytoplasm and trigger the ubiquitination of NRF2, leading to its degradation by the proteasome. Electrophilic or oxidative stress modifies KEAP1 conformation that liberates NRF2, allowing its nuclear translocation. Then, NRF2 heterodimerizes with a small protein MAF and binds the ARE (Antioxidant response element) sequences to exert its transcriptional function (Tonelli et al., 2018). Moreover, the KeratinoSens is an *in vitro* assay used to assess the sensitization potential of a chemical and is based on NRF2 transcription in a KC model, HaCaT (Emter et al., 2010). NRF2 controls allergic skin inflammation during the sensitization phase induced by a contact sensitizer like cinnamaldehyde (CinA)(El Ali et al., 2013). CinA is used in commercial food, cosmetics, and agriculture (Doyle and Stephens, 2019). Vallion et al. (2022) showed that CinA 100 μM induces the NRF2 pathway and has an anti-inflammatory effect in an *in vitro* KC model, KERTr cells.

NRF2 and NF-κB pathways interact with each other through a range of complex molecular interactions. The absence of NRF2 is associated with an exacerbation of NF-κB activity. NF-κB can have positive and negative effects on the expression of NRF2 target genes. For example, p65 can exert a negative effect on ARE-linked gene expression by different ways and HO-1 has a prominent role in NRF2-mediated NF-κB inhibition. In fact, the two pathways regulate the fine balance of cellular redox status and responses to stress and inflammation (Wardyn et al., 2015).

The first objective of this work was to study the mechanism of action of a QAC called ethylhexadecyldimethylammonium bromide (EHD) in human KC. EHD is classified in category 2 for skin irritation according to CLP (Classification, Labelling, Packaging) criteria^3^. But it has limited published research describing its sensitization potential. Then, it could be interesting to investigate its effects in human KC. The second objective was to study the potential effects of EHD on the antioxidant and anti-inflammatory effects of CinA 100 μM in KC (Vallion et al., 2022).

Then, KERTr cells were stimulated by EHD, CinA and a mixture of both. After stimulation, expression of NRF2-dependent-ARE-responsive genes and mRNA expression of pro-inflammatory cytokines that can be produced by KC during skin inflammation were measured. Proteins related to NRF2 pathway were also quantified. Our results suggested that, in KC, EHD did not induce detoxification *via* NRF2 pathway but triggered an inflammatory response. Moreover, the anti-inflammatory and antioxidant effects of CinA 100 μM were modulated by EHD when these two compounds were mixed. Our work involving invalidation of *NRF2* in KERTr cells showed the activity of NRF2 pathway on inhibition of EHD-induced inflammatory response in KC.

## Materials and methods

*Chemicals*: EHD (CAS No. 124-03-8), CinA (CAS No. 104-55-2) and Dimethylsulfoxide (DMSO; CAS No. 67-68-5) were purchased from Sigma-Aldrich (Saint-Quentin-Fallavier, France). EHD was dissolved in phosphate buffer saline (PBS; GIBCO, Illkirch, France) and CinA in DMSO. All vehicles were used at 0.1% final concentration in culture.

*Cell culture*: The human KC cell line CCD 1106 KERTr (ATCC CRL-2309™) was purchased from ATCC (Molsheim, France). For passing, the cells were trypsinized once a week (0.05% of trypsin-EDTA 1X; GIBCO) and seeded in a new culture flask at a cell density of 2 × 10^6^ cells/175 cm^2^ in a Keratinocyte-SFM serum-free medium (K-SFM; GIBCO), supplemented with bovine pituitary extract, epithelial growth factor (EGF), penicillin and streptomycin in concentrations of 0.05 mg/mL, 35 ng/mL, 10 U/mL and 10 μg/mL, respectively. The medium was renewed twice a week. The cells were cultured under standard cell culture conditions at a temperature of 37°C and 5% CO_2_.

*Invalidation of NRF2*: *NRF2* expression has been invalidated using sh RNA in KERTr cells as described by Vallion et al. (2022). Briefly, plasmid of expression for *NRF2* sh RNA (OriGene Technologies GmbH, Herford, Germany) was transferred into KERTr cell using lentiviral transduction. A scrambled sh RNA was used as control. 2 × 10^6^ cells at 50% of confluency were incubated overnight with 10 µg/mL of polybrene and 200 µg/mL of viral particles. Control KERTr cells (KERTr Sh Sc) and invalidated cells for *NRF2* (KERTr Sh *NRF2*) were sorted on a FACS Aria (BD Biosciences, Le Pont-de-Claix, France) based on the GFP (Green fluorescent protein) expression.

*Measurement of cytotoxicity*: On the day prior to the stimulation, KERTr cells were seeded into 24-well plates (4 × 10^5^ cells/well) in EGF-free culture medium. On the next day, solutions containing different concentrations of EHD (1–30 μM) were added for 24 h. Then, the cells were incubated with propidium iodide (PI; Invitrogen, Illkirch, France). Necrotic cells were stained by PI and were analyzed by flow cytometry using Attune NxT (ThermoFisher Scientific, Illkirch, France) and the FlowJo software (Becton, Dickinson & Company, Franklin Lakes, United States). Results were expressed as the percentage of living cells (PI^−^), and a concentration resulting in 70% cell viability of exposure (CV70) could be determined.

*Chemical exposure:* The cells were exposed to CinA 100 μM, EHD 10 μM, and a mixture containing both compounds at the same concentration for different exposure times to study the NRF2 pathway and inflammation in KC. Solutions of EHD and CinA were prepared in an EGF-free culture medium to get a concentration equal to their CV70. PBS (0.1%) was used as a negative control. During stimulation, the cells were cultured under standard cell culture conditions at 37°C and 5% CO_2_.

*Western blot analysis:* On the day before the stimulation, KERTr cells were seeded into 6-well plates (2 × 10^6^ cells/well) in an EGF-free culture medium. The solutions containing EHD (10 μM), CinA (100 μM), PBS (0.1%), and a mixture of EHD (10 μM) and CinA (100 μM) were added for different exposure times. At the end of each time of treatment, cultured KERTr cells were washed twice with cold PBS before lysis in lysis buffer containing 20 mM Tris HCl pH 7.4, 137 mM NaCl, 2 mM disodium EDTA pH 7.4, 1% Triton X-100, 2 mM sodium pyrophosphate, 10% glycerol, 25 mM β-glycerophosphate, 1 mM Na_3_VO_4_, 1 mM PMSF, 5 μg/mL aprotinin, 5 μg/mL leupeptin and 50 μg/mL pepstatin (Sigma-Aldrich). The homogenates were incubated for 20 min in ice and then centrifuged at 15,000 × g for 20 min at 4°C. Equal amounts of denatured proteins (30 µg) were loaded onto 10%SDS-PAGE gel (TCX Stain-Free FastCast, Bio-Rad, Marnes-la-Coquette, France) and transferred on polyvinylidene fluoride (PVDF) membranes (Bio-Rad). Membranes were blocked with a tri-buffered saline solution with 3% of bovin serum albumin for 1 h at room temperature. Then, they were incubated with primary antibodies anti-NRF2 (1/1000e, 16396-1-AP, Proteintech, Manchester, United Kingdom), anti-HO-1 (1/1000e, ab13248, Abcam, Paris, France), anti-NQO1 (1/1000e, ab28947, Abcam, Paris, France), at 4°C overnight. After several washings, membranes were incubated with secondary antibodies conjugated to Horseradish peroxidase (HPR) for 45 min at room temperature. To reveal primary antibodies anti-NRF2, anti-rabbit secondary antibodies were used. Primary antibodies anti-HO-1 and anti-NQO1 revelation was carried with anti-mouse secondary antibodies. Immunoreactive bands were detected by chemiluminescence using the ChemiDoc XRS + System (Bio-Rad Laboratories, Marnes la Coquette, France). Bands were quantified with Image Lab software and normalized with the total protein-loaded (Raffalli et al., 2018).

*RT-qPCR analysis:* The day before the stimulation, KERTr, KERTr Sh Sc, and KERTr Sh *NRF2* cells were seeded into 12-well plates (7.5 × 10^5^ cells/well). The solutions containing EHD (10 μM), CinA (100 μM), PBS (0.1%), or a mixture of EHD (10 μM) and CinA (100 μM) were added in wells for 2 h and 6 h. At the end of each time of treatment, cultured cells were trypsinized and centrifuged at 5,000 × g for 3 min at 4°C to get a cell pellet. Cells were then washed twice by centrifugation with cold PBS. Total RNA isolation was performed with PureLink™ RNA Mini (Invitrogen). Total RNA content was measured at 230, 260, and 280 nm by spectrophotometry for quantification and quality assessment. 500 ng total RNA were reverse-transcribed using the thermal cycler T100 (Bio-Rad) in a total-20 μL reaction mixture: 2.5 μM of Oligo (dT) (Promega, Charbonnières-les-Bains, France), 0.5 mM of deoxy-nucleotide triphosphate (dNTP; MP Biomedicals, Illkirch, France), 1 U/μL of Superscript IV Reverse Transcriptase (Invitrogen), 1 U/μL of Recombinant RNasin Ribonuclease Inhibitor (Promega), 5 mM of dithiothreitol (Invitrogen), 5 mM of reverse transcriptase buffer (Invitrogen). 1/20th of each generated cDNA was used for real-time PCR analysis, which was performed using the SYBR Green technology (Sso Advanced universal SYBR Green SuperMix, Bio-Rad) on thermal cycler CFX384 (Bio-Rad). As shown in Table 1, specific forward and reverse primers were used at a final concentration of 0.5 μM for the detection of the investigated genes *HMOX1, NQO1*, *IL1A*, *IL1B*, *IL6*, *CXCL8*, *IL23A*, *IL24*, *TNF*, *TSLP*, *ACTB* and *GAPDH* encoding, respectively, for HO-1, NQO1, IL-1α, IL-1β, IL-6, IL-8, IL-23 (subunit α), IL-24, TNF-α, TSLP, β-actin and Glyceraldehyde-3-phosphate dehydrogenase (GAPDH). Each sample was monitored in duplicate. The results were analyzed using the Bio-Rad CFX Manager software and were expressed by fold factor calculated by comparing the Ct values obtained from treated and untreated cells (PBS 0.1%) at the same time of exposure and corrected with *ACTB* and *GAPDH* expression.

**TABLE 1:** Sequences for primers used in RT-qPCR.

Gene	Forward primer	Reverse primer
*HMOX1*	5’ GGC​CTG​GCC​TTC​TTC​ACC​TT 3’	5’ GAG​GGG​CTC​TGG​TCC​TTG​GT 3’
*NQO1*	5’ GGG​CAA​GTC​CAT​CCC​AAC​TG 3’	5’ GCA​AGT​CAG​GGA​AGC​CTG​GA 3’
*IL1A*	5’ ATC​AGT​ACC​TCA​CGG​CTG​CT 3’	5’ TGG​GTA​TCT​CAG​GCA​TCT​CC 3’
*IL1B*	5’ ACA​GAC​CTT​CCA​GGA​GAA​TG 3’	5’ GCA​GTT​CAG​TGA​TCG​TAC​AG 3’
*IL6*	5’ TCA​ATG​AGG​AGA​CTT​GCC​TG 3’	5’ GAT​GAG​TTG​TCA​TGT​CCT​GC 3’
*CXCL8*	5’ TCT​CTT​GGC​AGC​CTT​CCT​GA 3’	5’ TGG​GGT​GGA​AAG​GTT​TGG​AG 3’
*IL23A*	5’ GTT​CCC​CAT​ATC​CAG​TGT​GG 3’	5’ TCC​TTT​GCA​AGC​AGA​ACT​GA 3’
*IL24*	5’ GCT​CTC​CGG​AAT​AGC​AGA​AAC 3’	5’ TCA​CAA​CTG​CAA​CCC​AGT​CA 3’
*TNF*	5’ CCT​CTC​TCT​AAT​CAG​CCC​TCT​G 3’	5’ GAG​GAC​CTG​GGA​GTA​GAT​GAG 3’
*TSLP*	5’ CAC​CGT​CTC​TTG​TAG​CAA​TCG 3’	5’ TAG​CCT​GGG​CAC​CAG​ATA​GC 3’
*ACTB*	5’ GGG​TCA​GAA​GGA​TTC​CTA​TG 3’	5’ GGT​CTC​AAA​CAT​GAT​CTG​GG 3’
*GAPDH*	5’ ACC​ACA​GTC​CAT​GCC​ATC​AC 3’	5’ TCC​ACC​ACC​CTG​TTG​CTG​TA 3’

*Statistical analysis:* The Mann-Whitney U-test was used. All data are presented as mean values ±SEM. Measurements were considered significant at a *p-value* ≤ 0.05 or ≤0.1 using GraphPad Prism (San Diego, United States).

## Results

A CV70 of EHD equal to 10 μM in KERTr cells was determined by flow cytometry (data not shown) and was used in this work. For CinA, a value of CV70 equal to 100 μM had already been determined in previous work. Moreover, the ability of CinA 100 μM to induce NRF2 pathway had been shown in KERTr cells (Vallion et al., 2022). The effect of the mixture EHD plus CinA on cell viability in KERTr cells was also investigated and did not induce different cell viability after treatment compared to EHD or CinA alone (Supplementary Figure S1).

### Study of NRF2 pathway in response to EHD and a mixture containing EHD and CinA

**FIGURE 1:**
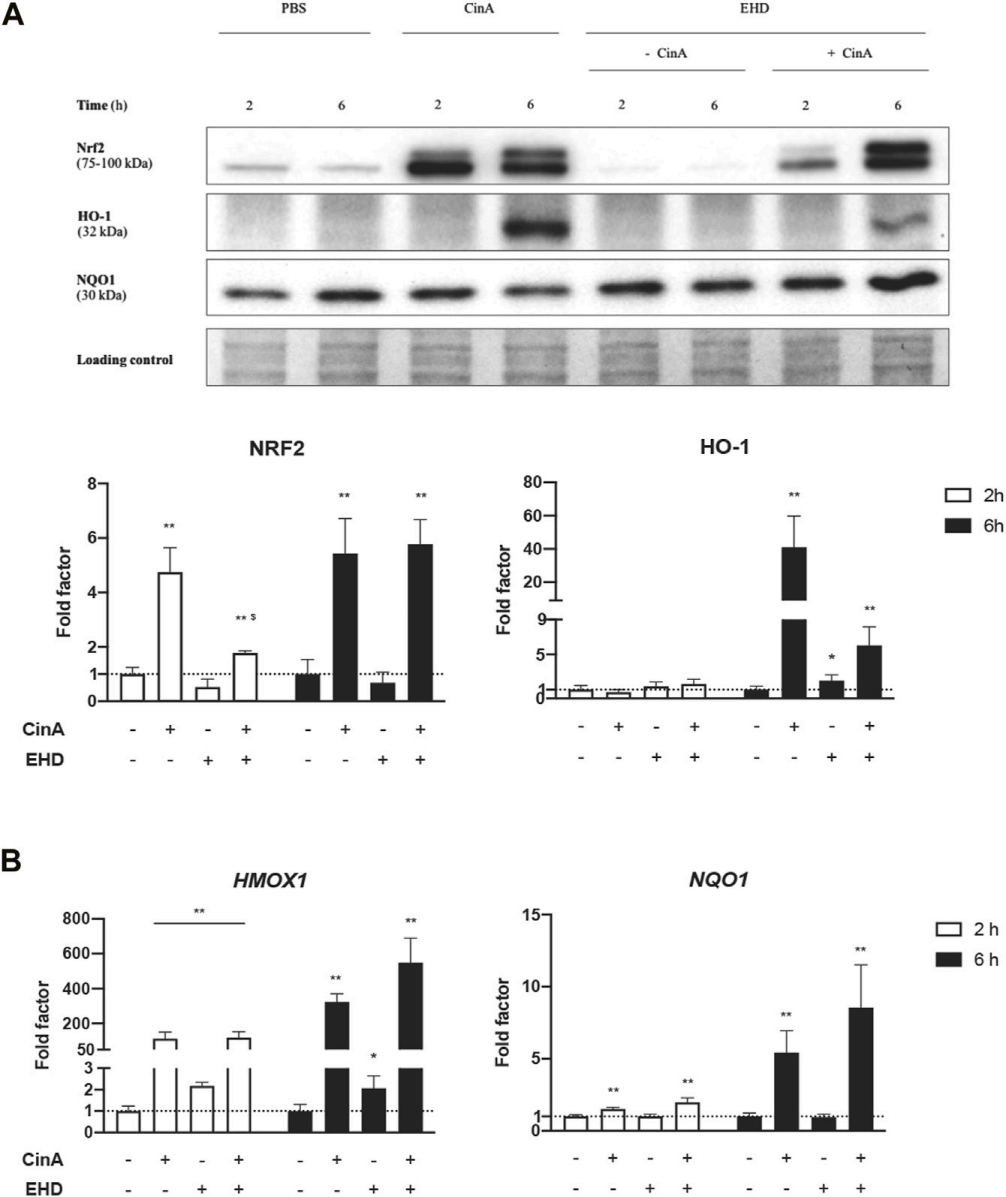
**EHD does not induce the NRF2 pathway in KERTr cells**. KERTr cells were treated for 2 and 6 h with CinA (100 μM), EHD (10 μM), a mixture containing CinA and EHD at the same concentrations, and PBS (0.1%) as vehicle control. **(A)** Effect of EHD on the expression of proteins of the NRF2 pathway. After treatment, protein expression of NRF2, HO-1, and NQO1 were quantified by Western blot with the Imagelab software (Bio-Rad). **(B)** Effect of EHD on the expression at gene level of two NRF2-dependent-ARE-responsive genes, *HMOX1* and *NQO1*. After treatment, the genic expression of *HMOX1* and *NQO1* were quantified by RT-qPCR. Results are expressed as the mean ± SEM of three duplicated independent experiments. * indicates significant differences from untreated control cells at the same time of stimulation (Mann-Whitney test, **p ≤ 0.05 and *p ≤ 0.1), and $ indicates significant differences between CinA and the mixture at the same time of stimulation (Mann-Whitney test, *p* ≤ 0.05).

KERTr cells were treated for 2 h or 6 h with EHD, CinA or a mixture of these two compounds. NRF2, HO-1 and NQO1 protein levels were measured by Western blot. Regarding EHD, no accumulation of NRF2 and a low expression of HO-1 (*p* ≤ 0.1) were quantified. Level of NRF2 protein was significantly increased following 2-h and 6-h treatment with CinA and the mixture (Figure 1A). However, after 2-h treatment, NRF2 expression was significantly lower with the mixture compared to CinA. Induction in HO-1 protein expression was shown following 6-h treatment with CinA or the mixture. Only a slight induction with EHD was quantified (*p* ≤ 0.1). This effect was weaker with the mixture. No change was noticeable concerning NQO1 protein expression.

We measured the mRNA expression of *HMOX1* and *NQO1* by RT-qPCR. Our results indicate that *HMOX1* mRNA expression was significantly increased after 2-h or 6-h treatment with EHD, but the measured values were too low to expect any concrete effect in the cell. Gene expression of both *HMOX1* and *NQO1* was significantly increased following 2-h and 6-h treatment with CinA and the mixture (Figure 1B).

### Induction of an inflammatory response by EHD

**FIGURE 2:**
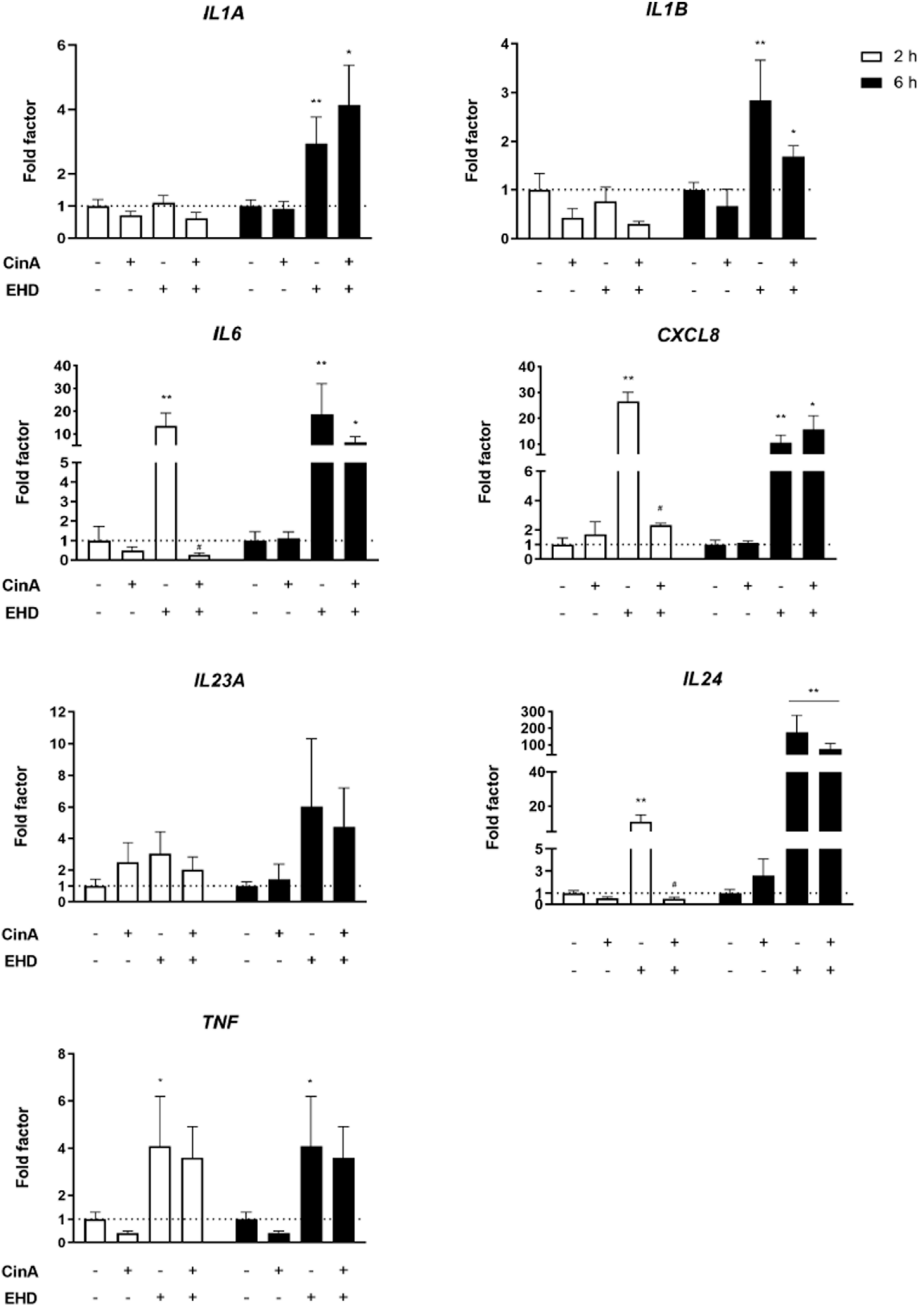
**EHD induces an inflammatory response in KERTr cells**. KERTr cells were treated for 2 and 6 h with CinA (100 μM), EHD (10 μM), a mixture containing CinA and EHD at the same concentrations, and PBS (0.1%) as vehicle control. After treatment, expression at gene level of *IL1A, IL1B, IL6, CXCL8, IL23A, IL24*, and *TNF* encoding, respectively, IL-1α, IL-1β, IL-6, IL-8, IL-23, IL-24, and TNF-α, were quantified by RT-qPCR and normalized with *ACTB* and *GAPDH* expression at gene level. Results are expressed as the mean ± SEM of three duplicated independent experiments. * indicates significant differences from untreated control cells (Mann-Whitney test, **p ≤ 0.05 and *p ≤ 0.1). # indicates significant differences between EHD and the mixture at the same time of stimulation (Mann-Whitney test, *p* ≤ 0.05).

After 2 and 6 h of incubation of KERTr cells with EHD, CinA, or the mixture of the two, mRNA expression of pro-inflammatory cytokines was measured by RT-qPCR. *IL24* mRNA expression was induced after 6-h treatment with EHD and the mixture (*p* ≤ 0.05). A higher expression was measured for *IL1A, IL1B, IL6* and *CXCL8* after 6-h treatment with EHD (*p* ≤ 0.05) and with the mixture (*p* ≤ 0.1). An increase in transcript expression of *TNF* was quantified after 2-h and 6-h treatment with EHD (*p* ≤ 0.1). Induction of mRNA expression was rapid and important for *IL6*, *CXCL8*, *IL24* (*p* ≤ 0.05) and *TNF* (*p* ≤ 0.1) in KERTr cells exposed to EHD since a high mRNA expression was quantified after 2-h treatment. Moreover, statistically significant inhibition of the effect of EHD by CinA on mRNA induction of some of these cytokines is noticeable in KERTr cells for the mixture as shown for *IL6*, *CXCL8,* and *IL24* following 2-h treatment (Figure 2). Ct values measured for *TSLP* transcript expression were not biologically relevant (data not shown).

### Inhibition of EHD-induced inflammatory response by NRF2 pathway

Since NRF2 has been shown to have anti-inflammatory properties (Suzuki and Yamamoto, 2017), invalidation of *NRF2* expression was carried out to study its potential inhibition on inflammatory response in KC induced by EHD. After stimulation of KERTr Sh *NRF2* and KERTr Sh Sc cells by EHD, CinA and the mixture, mRNA expressions of pro-inflammatory cytokines were investigated by RT-qPCR. Each experimental condition was compared to the control (KERTr Sh Sc with vehicle alone) and to its equivalent condition in each KERTr cell type (KERTr Sh *NRF2 versus* KERTr Sh Sc).

**FIGURE 3:**
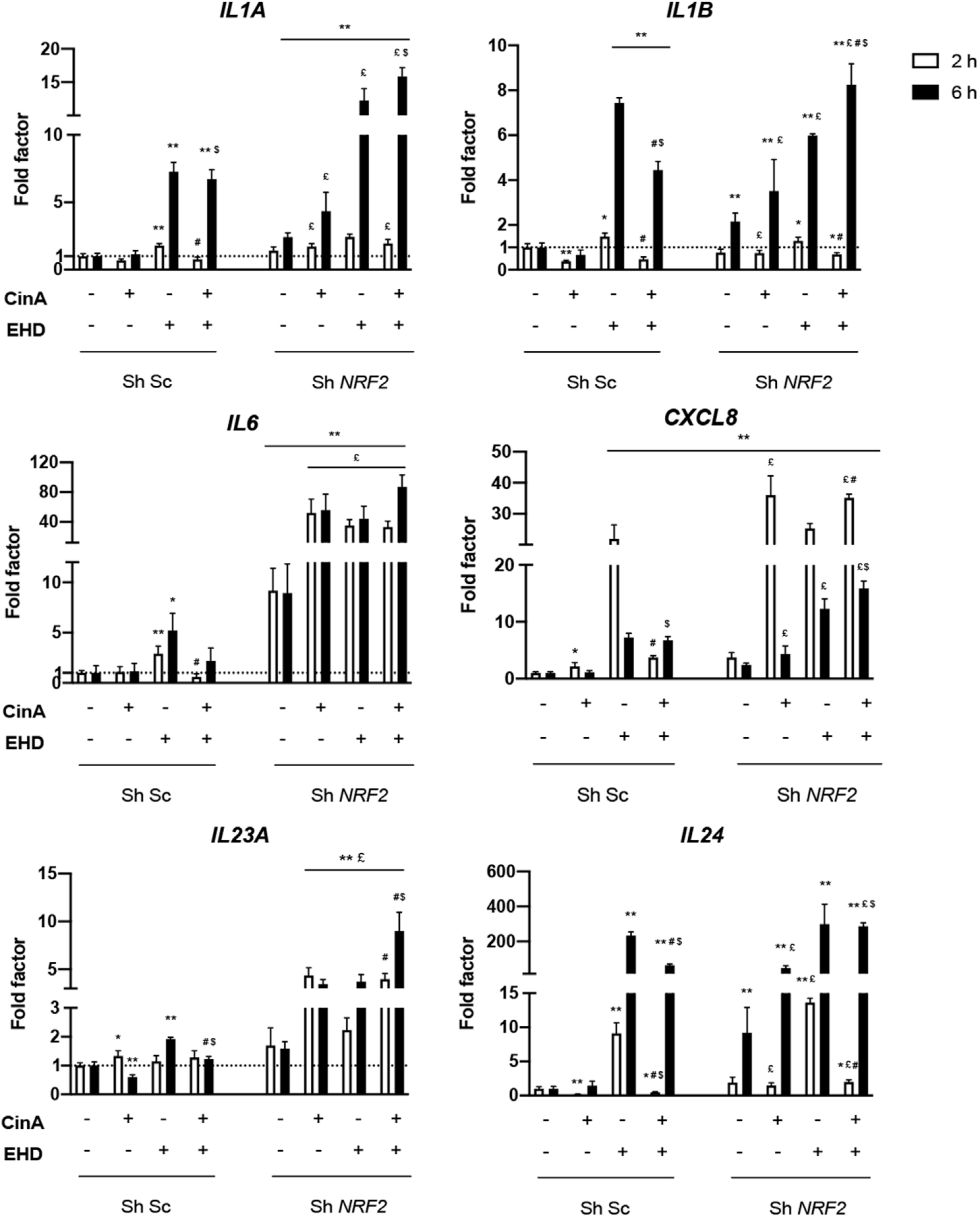
**NRF2 inhibits inflammation in KERTr cells exposed to EHD and the mixture of EHD and CinA**. Control KERTr cells (Sh Sc) and *NRF2*-invalidated KERTr cells (Sh *NRF2*) were treated for 2 and 6 h with CinA (100 μM), EHD (10 μM), a mixture containing CinA and EHD at the same concentrations and PBS (0.1%) as vehicle control. After treatment, expression at gene level of *IL1A, IL1B, IL6, CXCL8, IL23A*, and *IL24*, encoding, respectively, IL-1α, IL-1β, IL-6, IL-8, IL-23, and IL-24, were quantified by RT-qPCR and normalized with *ACTB* and *GAPDH* expression at gene level. Results are expressed as the mean ± SEM of three duplicated independent experiments. * indicates significant differences from untreated control Sh Sc KERTr cells (Mann-Whitney test, **p ≤ 0.05 and *p ≤ 0.1). £ indicates significant differences between Sh Sc and Sh *NRF2* for each condition at the same time of stimulation. $ indicates significant differences between CinA and the mixture, at the same time of stimulation, in the same type of cells. # indicates significant differences between EHD and the mixture, at the same time of stimulation, in the same type of cells (Mann-Whitney test, *p* ≤ 0.05).

Invalidation of *NRF2* in KERTr cells led to an induction of gene expression encoding IL-1α, IL-1β, IL-6, IL-8 and IL-24 at the steady state. After stimulation with EHD or the mixture, this induction was increased compared to the control cells. In KERTr Sh *NRF2 versus* KERTr control, EHD led to a higher expression of *IL24* after 2 h of treatment; *IL1A* and *CXCL8* after 6 h of treatment; and *IL6, IL23A* after both times of treatment. The rapid increase in transcript expression of *IL6*, *CXCL8* and *IL24* was again noticeable, but it was mainly higher in KERTr Sh *NRF2* cells. Surprisingly, the expression of *IL1B* was decreased after a 6-h treatment of EHD in KERTr Sh *NRF2* compared to KERTr control (Figure 3).

Concerning CinA treatment in KERTr Sh *NRF2,* an increased mRNA expression was quantified for *IL1B* and *IL24* after 6-h treatment; *IL1A*, *IL6*, *CXCL8* and *IL23A* after both times of treatment compared to control. In our study, CinA induced an inflammatory response in absence of *NRF2* whereas no inflammatory response could be measured in KERTr Sh Sc as described by Vallion et al., 2022.

In KERTr Sh *NRF2*, the mixture also caused an increase in *IL1A*, *IL6, CXCL8*, *IL23A* and *IL24* expressions after 2 and 6 h of treatment and in *IL1B* expression only after 6 h of treatment (Figure 3). Hence, it implies an inhibitory role of *NRF2* on the expression of these genes involved in inflammation and then, on inflammatory response induced by the tested compounds. For *TNF* gene, there were lots of variabilities making any conclusion difficult (data not shown).

After 2-h treatment, a significant decrease in *IL1B* and *IL24* expression with the mixture compared to EHD was measured in both types of KERTr cells (Figure 3). Contrary to normal KERTr cells, no difference between the mixture and the compound alone was measured in KERTr Sh *NRF2* cells for *IL6* expression. For *IL1B* expression in KERTr Sh *NRF2*, after 6 h of treatment, a higher expression can be highlighted with the mixture than EHD or CinA alone.

After 2-h treatment, *CXCL8* expression was lower with the mixture than with EHD in control KERTr cells whereas it was higher in *NRF2*-invalidated KERTr cells. After 6 h of treatment, the mixture (CinA + EHD) induced a higher expression of *CXCL8* than CinA alone. Regardless of the stimulation time, the mixture induced more *IL23A* mRNA expression in KERTr Sh *NRF2* cells compared to EHD. For the comparison with CinA, only the 6 h time point showed a difference with the mixture.

As in normal KERTr cells, *IL24* expression in KERTr Sh *NRF2* cells was decreased with the mixture compared to EHD after 2-h treatment and was increased compared to CinA after 6-h treatment even if invalidation of *NRF2* allowed induction of *IL24* expression by CinA (Figure 3).

## Discussion

Contact dermatitis (CD) can be allergic (ACD) or non-allergic, like irritant contact dermatitis (ICD). These inflammatory eczematous skin diseases have different pathophysiological mechanisms. In both cases, danger signals such as DAMP (Damage-associated molecular pattern) and ROS (reactive oxygen species) generated by KC can promote skin inflammation and recruitment of innate immune cells into the epidermis.

ICD is directly caused by the toxic effects of the compound on skin cells leading to unspecific immune responses. Its mechanism is not totally understood, and could imply the disorganization of the lipid layers of cell membranes and the damage of epidermal barrier proteins, *e.g.,* involucrin, filaggrin, or keratin. To promote an ICD, repetitive applications of some irritants may be necessary (Scheinman et al., 2021).

ACD requires the formation of an antigenic complex composed of the hapten and a self-protein (Karlberg et al., 2008). The formation of this neo-antigen depends on different factors, including the hapten’s intrinsic properties and the exposure conditions, i.e., the dose, frequency, duration, vehicle, and formulation of the hapten. The sensitization phase can be induced after a first exposure to a contact allergen without symptoms. Then, DC in the skin are activated and can migrate to promote an adaptive immune response in the draining lymph node. Concerning compounds with weak or very weak sensitizing properties, the sensitization needs to be fully characterized (Scheinman et al., 2021).

To identify contact allergens, most studies focus on KC and DC (Martin, 2012). Indeed, KC are the first cells in contact with the irritant or contact allergens and are considered very active sentinel cells, which are involved in a wide range of inflammatory skin disorders (Jiang et al., 2020).

Some studies have shown that contact sensitizers induce the NRF2 pathway in KC and DC, whereas skin irritants do not (Ade et al., 2009; Vandebriel et al., 2010). *In vivo* experiments demonstrated that the NRF2 pathway, activated by contact sensitizer, including CinA, was involved in contact sensitizer-induced inflammation control (El Ali et al., 2013; Vallion et al., 2022).

In our work, we studied the effect of a QAC called EHD on the NRF2 pathway in a cellular model of human KC. Our results showed that EHD did not efficiently promote NRF2 expression and its related genes, *HMOX1* and *NQO1,* in KERTr cells. As expected, CinA 100 μM could induce the NRF2 pathway (Figure 1) (Vallion et al., 2022). That suggests that EHD may not act as a contact sensitizer in KC. However, it cannot be excluded the possibility that EHD may be a weak allergen like it had been suggested in the controversial case of lanolin (Lee and Warshaw, 2008).

Skin irritation is characterized by inflammation in the skin. It has a known role in ACD, both in the sensitization and elicitation phases (Smith et al., 2002). Moreover, when the skin is readily irritated, it seems more susceptible to contact sensitization (Elsner and Burg, 1993). We, therefore, studied the potential inflammatory effect of EHD in KERTr cells. We showed that EHD triggered an inflammatory response at the transcriptional level since it induced the expression of genes encoding pro-inflammatory cytokines.

IL-6 is a cytokine whose expression can be induced after the application of irritants on the skin. It has a prominent role in the chemoattraction of neutrophils and T-cells (Lee H. Y. et al., 2013). However, *in vivo* studies in mice demonstrated the anti-inflammatory effects of IL-6, which depended on the nature of the irritant (Lee E. G. et al., 2013). IL-8 is involved in neutrophil recruitment and activation during skin inflammation (Harada et al., 1994). Neutrophils have an essential role in the sensitization and elicitation phases of contact hypersensitivity, an animal model of human ACD. Indeed, neutrophils have a role in DC induction and migration (Weber et al., 2015). Therefore, EHD could promote these events when applied to the skin.

In our study, we measured an increase in *IL1B* expression in KERTr cells, but we could not show a higher production of IL-1β in the supernatants (Supplementary Figure S2). The NALP3 (nucleotide-binding oligomerization domain (NOD)–like receptor family domain-containing protein 3) inflammasome allowing the generation of the active form of IL-1β in KC has been demonstrated as a key regulator of innate immunity in contact hypersensitivity (Watanabe et al., 2007). It suggests that EHD could not activate the NALP3 inflammasome in KERTr cells.

Because NF-κB is a central mediator of pro-inflammatory gene induction (Juráňová et al., 2017), we also investigated its related pathway. After EHD short-term treatments in KERTr cells, it could be noted a decrease in IκBα expression and an increase in phospho-p65 expression by Western blot (Supplementary Figure S3). These signs occurred for NF-κB pathway activation (Juráňová et al., 2017). It can imply that the induction of NF-κB pathway by EHD was the source of the inflammatory response measured at the transcriptional level in KERTr cells. However, the study of inflammatory response at the protein level was more ambiguous because of the high variabilities between experiments. Nevertheless, EHD appeared to trigger an increase in protein expression of IL-6 and IL-8 (Supplementary Figure S2). IL-1β and TNF-α are two *stimuli* of NF-κB pathway and they could not be clearly found after 24 h of EHD treatment in cell supernatants (Supplementary Figure S2) (Juráňová et al., 2017). Consequently, other *stimuli* such as ROS and DAMP generated by KC exposed to EHD must be sought. If EHD causes oxidative stress in KC, the absence of induction of the NRF2 pathway can result in ROS overproduction which could stimulate the NF-κB pathway.

Lee et al. (2019) have highlighted the importance of studying the skin sensitization potentials of chemical mixtures that humans could be occupationally or environmentally exposed to, *e.g.,* DDAC has a mitigated sensitization potential when it is mixed with an increasing amount of ethylene glycol. Nilzen and Wikstrom, (1955) had already demonstrated sensitization to chromium salts and nickel in guinea pigs which could only occur in the presence of a known skin irritant, sodium lauryl sulfate (SLS). We showed that a mixture of EHD (10 μM) and CinA (100 μM) induced the NRF2 pathway in KERTr cells. More interestingly, NRF2 expression after 2-h treatment with the mixture was diminished compared to CinA treatment (Figure 1). Then, EHD has a rapid and transient inhibitory effect on NRF2 protein level which could result in an inhibition of the antioxidant effect of CinA in KC. However, according to our work, this potential modulation in the cell would occur *via* different ways other than inhibition of HO-1, *HMOX1* or *NQO1* expression. Besides, our studies in KERTr cells revealed that the mixture induced the expression of genes encoding pro-inflammatory cytokines. However, compared with EHD alone, we demonstrated that *IL6*, *CXCL8,* and *IL24* expression was lower. This trend was followed by decreased expression of IL-6 and IL-8 in the supernatants (Supplementary Figure S2). This inhibition may be the result of the anti-inflammatory effect of CinA. Indeed, it was shown that CinA exerts its anti-inflammatory effect *via* its ability to induce the NRF2 pathway (Vallion et al., 2022). Then, thanks to *NRF2*-invalidated KERTr cells, we demonstrated an apparent global intensification of the inflammatory response at the transcriptional level when *NRF2* was repressed compared to the control (Figure 3). These findings underlined the efficient anti-inflammatory role of NRF2, even at its basal activity. It is described in the literature that NRF2 and NF-κB pathways interact widely with each other (Wardyn et al., 2015). As NRF2 displayed a repressive activity on inflammatory response triggered by EHD or the mixture in KERTr cells, the transcription factor could inhibit the mRNA expression of pro-inflammatory cytokines by interacting negatively with the NF-κB pathway.

Consequently, the presence of EHD might adversely modulate the skin sensitization potential of CinA in KC. However, we did not demonstrate that EHD was a skin irritant like SLS. Other studies have suggested that irritants may become allergens when exposed to damaged skin (Engebretsen and Thyssen, 2016). Because QAC is found in everyday products with other compounds, it would be interesting to investigate the sensitization potential of QAC in the presence of skin irritants in a cellular model of KC.

Besides, neuromuscular blocking agents (NMBA) are drugs used during anesthesia and are also QAC. They are known to provoke unexpected anaphylactic shock on first injection. The origin of how those patients get sensitized to NMBA remains unclear. A cross-reaction between NMBA and other QAC after a prior skin sensitization of QAC *via* everyday products is suspected to be the cause of these anaphylactic shocks. Then, more investigation about the effects of QAC on KC may also bring new evidence concerning this assumed link (Peyneau et al., 2022).

## Data Availability

The original contributions presented in the study are included in the article/Supplementary Material, further inquiries can be directed to the corresponding author.

## Footnotes

1 CAS No. 8001-54-5.

2 CAS No. 7173-51-5.

3 Notified classification and labelling according to CLP criteria.

## Abbreviations

ACD, allergic contact dermatitis; CinA, cinnamaldehyde; CV, cell viability; DAMP, damage-associated molecular patterns; DC, dendritic cells; EHD, ethylhexadecyldimethylammonium bromide; KC, Keratinocytes; NMBA, Neuromuscular blocking agents; NRF2, nuclear factor (erythroid-derived 2)-like 2; QAC, quaternary ammonium compounds; ROS, reactive oxygen species.
