# Supplementary material for: Ethylhexadecyldimethylammonium bromide, a quaternary ammonium compound, controls inflammatory response through NRF2 pathway in a human immortalized keratinocyte cell line
Source: Front Toxicol. 2023 Apr 6;5:1132020. doi: 10.3389/ftox.2023.1132020 (PMC10117438; doi:10.3389/ftox.2023.1132020)
Supplement: Supplementary file 1 [file DataSheet2.PDF]

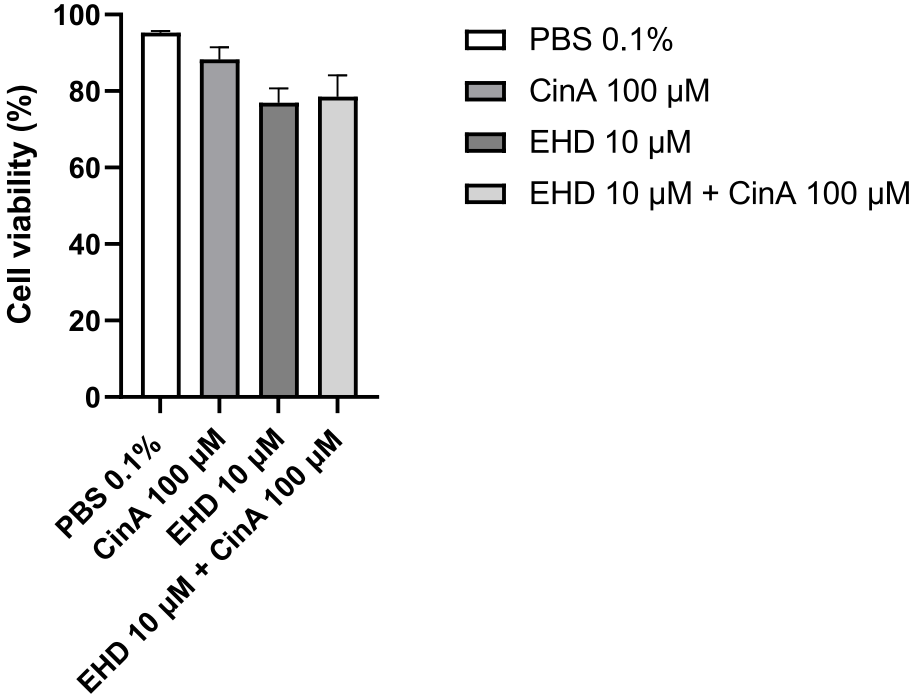

Supplementary Figure 1. There is no difference in cell viability in KERTr cells after treatment with the mixture EHD plus CinA compared to EHD and CinA alone. On the day prior to the stimulation, KERTr cells were seeded into 24-well plates (4 x 10^5 cells/well) in EGF-free culture medium. On the next day, solutions containing EHD (10 µM), CinA (100 µM) or a mixture of CinA (100 µM) and EHD (10 µM) were added for 24 h. Then, the cells were incubated with propidium iodide (PI; Invitrogen, Illkirch, France). Necrotic cells were stained by PI and were analyzed by flow cytometry using Attune NxT (ThermoFisher Scientific, Illkirch, France) and the FlowJo software (Becton, Dickinson & Company, Franklin Lakes, USA). Results were expressed as the percentage of living cells (PI^-).

The results were expressed as the mean ± SEM of three duplicated independent experiments.

PBS is the vehicle control of EHD.

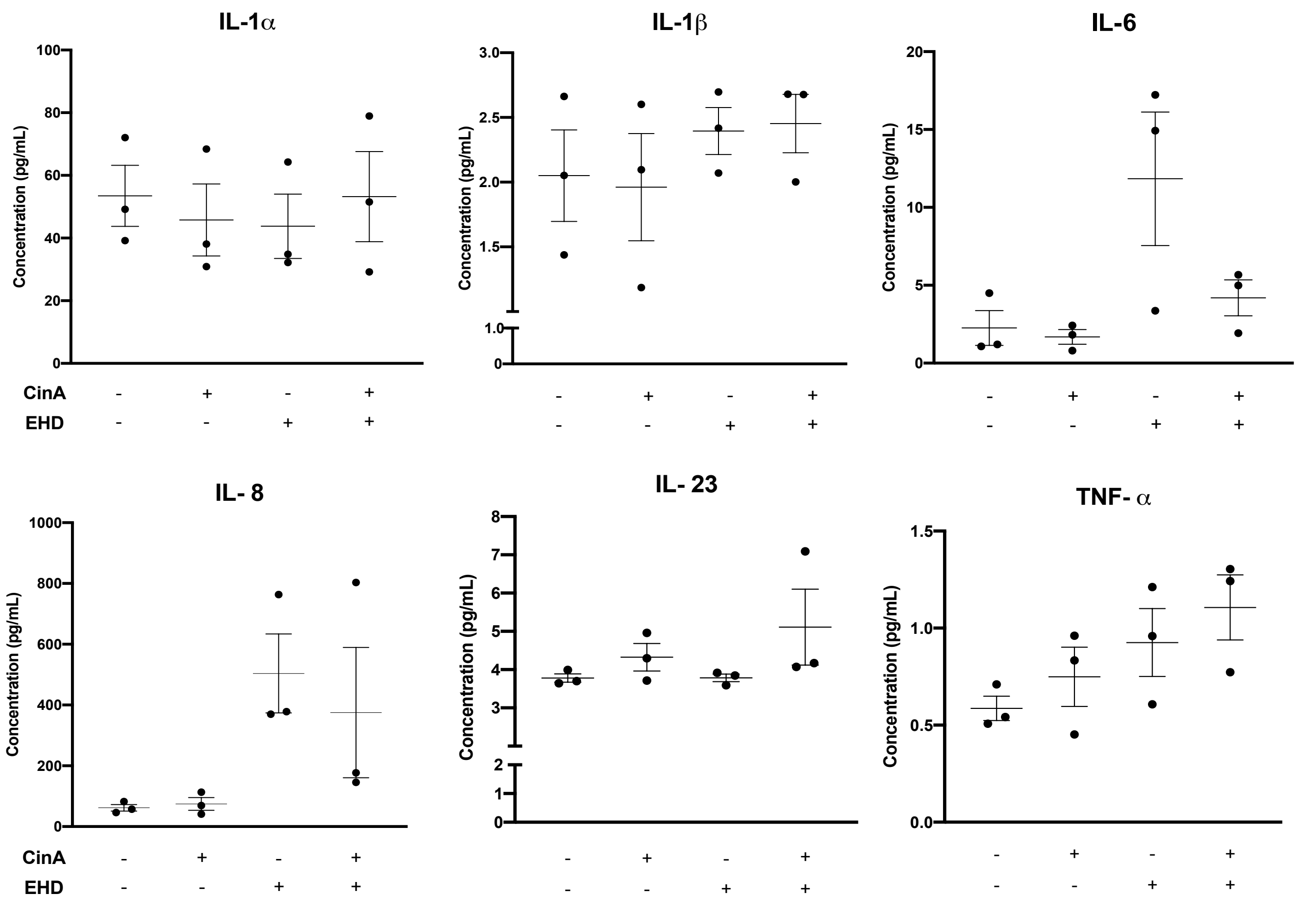

Supplementary Figure 2. **EHD may induce secretion of some pro-inflammatory cytokines by KC**. On the day prior to the stimulation, KERTr cells were seeded into 12-well plates (7.5 x 10^5 cells/well) in supplemented EGF-free K-SFM. The solutions containing EHD (10 μM), CinA (100 μM), PBS (0.1 %) and a mixture of EHD (10 μM) and CinA (100 μM) were added for different exposure times. After, the culture supernatants were centrifuged at 12,000xg for 10 min at 4°C. The supernatants were collected. Meso Scale Discovery (MSD, Meso Scale Diagnostics, Rockville, USA) U-PLEX® Biomarker Group 1 (Human) assay was performed on supernatants according to the manufacturer's instructions. MSD 96-well plate was read on MSD MESO® QuickPlex SQ 120 Instrument to measure in duplicate IL1-α, IL1-ß, IL-6, IL-8, IL-23 and TNF-α protein expression. The results were analyzed using the software MSD Discovery Workbench (Meso Scale Discoveries, Rockville, USA) and were expressed as the mean ± SEM of three duplicated independent experiments.
PBS is the vehicle control of EHD.

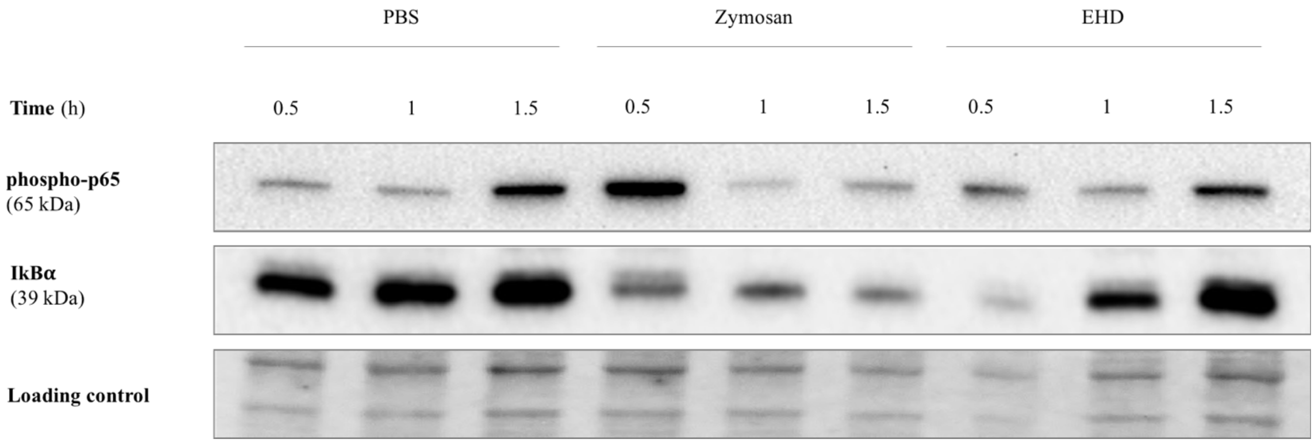

Supplementary Figure 3. EHD may activate inflammatory response via NF-κB pathway activation. KERTr cells were seeded into 6-well plates (2 x 10^6 cells/well) in supplemented EGF-free K-SFM. The solutions containing EHD (10 µM), PBS (0.1 %) and zymosan (20 µg/mL; Sigma-Aldrich) were added for 0.5 h, 1 h and 1,5 h. At the end of each time of treatment, cultured KERTr cells were washed twice in cold PBS before lysis in lysis buffer (20 mM Tris HCl pH 7.4, 137 mM NaCl, 2 mM disodium EDTA pH 7.4, 1% Triton X-100, 2 mM sodium pyrophosphate, 10% glycerol, 25 mM β-glycerophosphate, 1 mM Na_3VO_4, 1 mM PMSF, 5 µg/mL aprotinin, 5 µg/mL leupeptin and 50 µg/mL pepstatin). All these products are from Sigma-Aldrich. The homogenates were incubated for 20 min in ice and then centrifuged at 15,000g for 20 min at 4°C. Equal amounts of denatured proteins were loaded onto 10%SDS-PAGE gel (TCX Stain-Free FastCast, Bio-Rad, Marnes-la-Coquette, France) and transferred on PVDF membrane (Bio-Rad). Membranes were successively blocked, incubated with primary antibodies anti-Nrf2 (1/1000e, 16396-1-AP, Proteintech, Manchester, United Kingdom), anti-HO-1 (1/1000e, ab13248, Abcam, Paris, France), anti-NQO1 (1/1000e, ab28947, Abcam, Paris, France), and then with secondary antibodies conjugated to Horseradish peroxidase (HRP). Immunoreactive bands were detected by chemiluminescence using the ChemiDoc XRS+ System (Bio-Rad Laboratories, Marnes la Coquette, France). Bands were quantified with ImageLab software and normalized with the total protein loaded (Raffalli et al., 2018).
PBS is the vehicle control of EHD and zymosan is a positive control of the experiment.

References

Raffalli, C., Clouet, E., Kuresepi, S., Damiens, M.-H., Lepoittevin, J.-P., Pallardy, M., et al. (2018). Editor's Highlight: Fragrance Allergens Linalool and Limonene Allylic Hydroperoxides in Skin Allergy: Mechanisms of Action Focusing on Transcription Factor Nrf2. *Toxicological Sciences* 161, 139–148. doi: 10.1093/toxsci/kfx207.
