# Supplementary material for: Ethylhexadecyldimethylammonium bromide, a quaternary ammonium compound, controls inflammatory response through NRF2 pathway in a human immortalized keratinocyte cell line
Source: Front Toxicol. 2023 Apr 6;5:1132020. doi: 10.3389/ftox.2023.1132020 (PMC10117438; doi:10.3389/ftox.2023.1132020)
Supplement: Supplementary file 2 [file DataSheet1.PDF]

Prof. Saadia Kerdine-Römer
saadia.kerdine-romer@universite-paris-saclay.fr
INSERM UMR-S 996 - Inflammation, Microbiome & Immunosurveillance
Université Paris-Saclay, Faculté de Pharmacie - 17 av des Sciences –
F-91400 Orsay
Phone: +33 (0) 1 8000 6467

Ref: 100% discount with code DSC-12035242720PRD

Orsay, December 26th, 2022

Dear editors,

I would like to thank you for the invitation to submit an article to your special issue, "Women in Immunotoxicology." You will find attached the manuscript entitled " Ethylhexadecyldimethylammonium bromide, a quaternary ammonium compound, induces an inflammatory response and modulates the effect of a skin sensitizer on NRF2 pathway in Human Keratinocytes " that we would like to submit for publication in Frontiers in Toxicology.

Our group is interested in the role of Nrf2 in innate immunity, particularly in skin allergy. For the last three years, we have focused our study on keratinocytes, the main cells in the epidermis.

In this work, we study the mechanism of action of a quaternary ammonium compound called ethylhexadecyldimethylammonium bromide (EHD) in human keratinocytes cells (KERTr cell line, a primary human cell line immortalized). EHD is classified in category 2 for skin irritation according to CLP (Classification, Labelling, Packaging) criteria labeling (Notified classification and labelling according to CLP criteria, 2022). But it needs more published research describing its sensitization potential. We have therefore studied the effect of EHD on the NRF2 pathway in a KERTr cell.

As quaternary ammonium compounds are found in everyday products with other compounds, we also studied the mixture of EHD with a contact sensitizer at low concentrations, not inducing inflammatory responses (CinA 100 μM, Vallion *et al.*, Antioxydant, 2022).

Our results suggested that, in KC, EHD did not induce detoxification via the NRF2 pathway but triggered an inflammatory response. Moreover, the anti-inflammatory and antioxidant effects of CinA 100 μM were modulated by EHD when these two compounds were mixed. Our work involving invalidation of NRF2 in KERTr cells showed the activity of the NRF2 pathway on inhibition of EHD-induced inflammatory response in KC.

Thank you for your time and for considering our manuscript.

Sincerely yours,

Prof. Saadia Kerdine-Römer, PhD
